# 表皮生长因子受体突变细胞系H1650耐药机制探讨

**DOI:** 10.3779/j.issn.1009-3419.2012.12.02

**Published:** 2012-12-20

**Authors:** 瑞丽 韩, 小丽 王, 殿胜 钟, 娟 赵, 哲 陈, 琳琳 孙, 竞 王, 金棒 张

**Affiliations:** 1 300052 天津，天津医科大学总医院呼吸科 Department of Respiratory Medicine, Tianjin Medical University General Hospital, Tianjin 300052, China; 2 300052 天津，天津医科大学总医院肿瘤科 Department of Medical Oncology, Tianjin Medical University General Hospital, Tianjin 300052, China; 3 天津市肺癌研究所 Tianjin Lung Cancer Institute, Tianjin Medical University General Hospital, Tianjin 300052, China

**Keywords:** 肺肿瘤, 表皮生长因子受体, 突变, Erlotinib耐药, Lung neoplasms, Epidermal growth factor receptor, Mutation, Erlotinib resistance

## Abstract

**背景与目的:**

表皮生长因子受体（epidermal growth factor receptor, EGFR）高表达和突变与40%左右的肺癌有关，已成为靶向治疗药物研究热点；随着Gefitinib和Erlotinib作为EGFR酪氨酸激酶抑制剂（tyrosine kinase inhibitor, TKI）代表药物应用于临床，继而产生的耐药现象亦成为临床一大难题，部分耐药机制仍不清楚。本研究探讨非小细胞肺癌（non-small cell lung cancer, NSCLC）细胞系H1650耐药机制。

**方法:**

选用real-time RT-PCR检测EGFR野生型NSCLC细胞系中EGFR mRNA表达水平；MTT检测癌细胞对Erlotinib的药物敏感性；Western blot检测*EGFR*突变NSCLC细胞系突变情况和Erlotinib及PI3K抑制剂（LY294002）对*EGFR*突变型NSCLC细胞下游信号蛋白磷酸化水平的影响。

**结果:**

*EGFR*野生型细胞系中，EGFR mRNA表达水平高低不一，但均对Erlotinib耐药；*EGFR*突变型细胞系中，HCC827和H1650为同种突变类型，HCC827对Erlotinib敏感，H1650则相对耐药；检测显示，H1650细胞中PTEN表达缺失，给予Erlotinib和LY294002处理后，HCC827中p-AKT明显被抑制，但H1650中p-AKT下调不明显。

**结论:**

在NSCLC细胞系中，Erlotinib药物敏感性与EGFR的mRNA表达高低无关，但与EGFR的突变类型有关；H1650对Erlotinib相对耐药可能与PTEN缺失导致的p-AKT持续活化有关。

## Abstract (en)

**Background and objective:**

Epidermal growth factor receptor (EGFR) overexpression and mutations were existed in more than 40% of the lung cancer, and it's the one of molecular targets in clinical treatment. But the EGFR tyrosine kinase inhibitors (TKI)-resistance is becoming a challenging clinical problem as following the application of EGFR-TKIs, Gefitinib or Erlotinib. However, the mechanistic explanation for resistance in the some cases is still lacking. Here we researched the resistance mechanism of H1650 cells.

**Methods:**

Using real-time RT-PCR to analyze the EGFR mRNA expression level in EGFR wild-type non-small cell lung cancer (NSCLC) cells; MTT analysis detected the cytotoxicity for NSCLC cells to Erlotinib; Western blot analysis examined the mutant situations and the downstream signaling protein phosphorylation level in *EGFR*-mutant NSCLC cells with the treatment of Erlotinib or/and PI3K inhibitor, LY294002.

**Results:**

In the EGFR wild-type NSCLC cells, the expression level of EGFR mRNA varied dramatically and all the cells showed resistant to Erlotinib; In the *EGFR*-mutant cells, HCC827 and H1650 (the same activating-mutation type), HCC827 cells were Erlotinib-sensitive as well as H1650 demonstrated primary relative resistance. Western blot analysis showed the loss of PTEN and the p-AKT level was not inhibited with the treatment of Erlotinib or/and LY294002 in H1650 cells, while HCC827 cells were no PTEN loss and definitively decrease of p-AKT level.

**Conclusion:**

EGFR wild-type NSCLC cells were resistant to Erlotinib no matter of how EGFR mRNA expression level. *EGFR*-activating mutations correlated with responses to Erlotinib. The PTEN loss and activation of AKT signaling pathway contributed to Erlotinib resistance in *EGFR*-mutant NSCLC cell line H1650.

肺癌主要分为非小细胞肺癌（non-small cell lung cancer, NSCLC）和小细胞肺癌（small cell lung cancer, SCLC），其中80%-85%为NSCLC。尽管现代医学的诊疗技术已经有了相当的进展，采取了手术为主、放化疗等综合治疗手法，肺癌总的5年生存率仍只有13%-15%^[1]^。研究^[2, 3]^发现，表皮生长因子受体（epidermal growth factor receptor, EGFR）在50%-90%的NSCLC患者中高表达，参与肿瘤的血管新生、迁移和粘附过程，其扩增和突变已被认为是肺部肿瘤发生的主要机制之一。厄洛替尼（Erlotinib, Tarceva）是EGFR酪氨酸激酶抑制剂（tyrosine kinase inhibitor, TKI），通过特异性结合在EGFR胞内酪氨酸激酶区域抑制EGFR活化而发挥抗瘤作用^[4]^。本研究探讨了NSCLC细胞系*EGFR*基因的表达水平及其抑制剂Erlotinib对NSCLC细胞系的毒性作用。

## 1 材料与方法

### 1.1 材料

人NSCLC细胞系A549、H460、H157、H1299、H1792、CALU-1、H1650、H1975和HCC827购自美国模式培养物集存库（American Type Culture Collection, ATCC）。RPMI-1640培养基、新生小牛血清购于GIBCO公司；Trizol Reagent购自美国Invitrogen公司；反转录试剂、SYBR Premix Ex Taq购自TAKARA公司，MTT试剂盒购于美国Promega公司，p-AKT、p-ERK、AKT、ERK、PTEN抗体均购于美国Cell Signaling Technology公司，GAPDH抗体购于美国Santa Cruz公司，二抗HRP-羊抗兔IgG、二抗羊抗鼠IgG购于北京中杉生物技术有限公司，PVDF膜购于美国Amersham Biosciences。

### 1.2 方法

#### 1.2.1 细胞培养

用含10%小牛血清的RPMI-1640培养基、100 U/mL青霉素、100 U/mL链霉素配制成1640完全培养基，37 ℃、5%CO_2_、饱和湿度的培养箱中传代培养，0.125%胰酶消化传代，3 d-4 d传一代。所有实验均采用对数生长期细胞。

#### 1.2.2 Real-time RT-PCR

用Trizol试剂提取处于对数生长期细胞总RNA，反转录mRNA为cDNA，使用real-time RT-PCR技术检测基因的表达情况。PCR引物序列为：EGFR：Forward：GCGTTCGGCACGGTGTATAA；Reverse：GGCTTTCGGAGATGTTGCTTC；参照基因GAPDH：Forward：GGAGTCAACGGATTTGGTCG；Reverse：CTTGATTTTGGAGGGATCTCG，扩增长度为240 bp。PCR扩增体系20 μL，第一步：预变性95 ℃、30 s、1个循环，第二步：PCR反应：95 ℃、5 s，60 ℃、34 s，40个循环。以GAPDH为内参照，2^-ΔΔCT^法计算EGFR基因表达差异。

#### 1.2.3 MTT法检测细胞存活率

取对数生长期细胞，常规胰酶消化制成单细胞悬浮液，以每孔约5×10^3^个细胞接种于96孔板，每孔200 μL，培养过夜后弃去原液，分别加入终浓度为1×10^-3^ μM/L、1×10^-2^ μM/L、1×10^-1^ μM/L、1 μM/L、10 μM/L和20 μM/L的Erlotinib，对照组加入等量的培养基，每组设4个复孔，72 h后进行MTT试验，并绘制细胞生长曲线。

#### 1.2.4 Western blot方法

按照文献^[5]^，提取细胞总蛋白，BCA法测定蛋白浓度，取50 µg蛋白上样，十二烷基硫酸钠-聚丙烯酰胺凝胶电泳，转膜、封闭，一抗4 ℃过夜，二抗室温1 h，使用ECL化学发光试剂工作液进行蛋白信号检测，GAPDH作为蛋白加样对照。

## 2 结果

### 2.1 *EGFR*野生型NSCLC细胞系中EGFR mRNA表达水平及与Erlotinib细胞毒性相关性的研究

#### 2.1.1 *EGFR*野生型NSCLC细胞系中EGFR mRNA表达水平

利用real-time RT-PCR技术检测了6种*EGFR*野生型NSCLC细胞系中EGFR mRNA表达水平。实验结果显示（图 1），H157表达水平最低；A549和CALU-1表达水平相对较低；H460和H1792表达水平较高；H1299表达水平最高，约为H157的2, 621倍，为EGFR高表达的细胞系。

**1:**
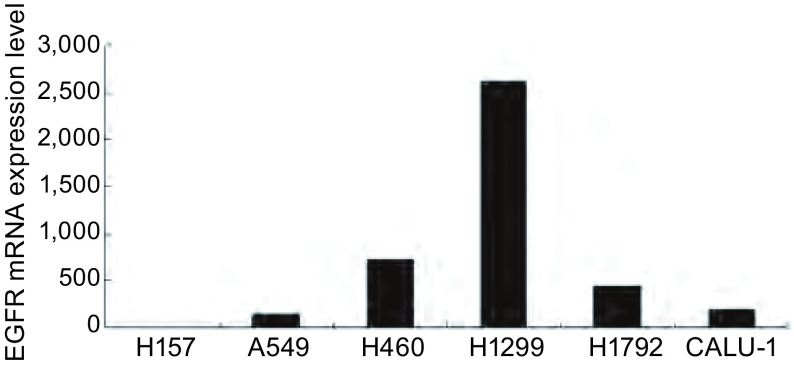
*EGFR*野生型NSCLC细胞系中EGFR mRNA表达水平 The epidermal growth factor receptor (EGFR) mRNA expression level in *EGFR* wild-type non-small cell lung cancer (NSCLC) cells

#### 2.1.2 Erlotinib对上述细胞系的毒性作用

6株细胞系分别在7个浓度梯度的Erlotinib培养液中培养72 h，利用MTT方法检测细胞毒性，结果显示（图 2）：随着药物浓度的倍增，Erlotinib对各细胞系的生长抑制作用并没有明显增强，均表现为明显的耐药性。其中在1 μΜ Erlotinib的浓度，H157细胞系的生长抑制率约为10%，A549和Calu-1的生长抑制率约为0，H460和H1792细胞系的生长抑制率分别约为12%和15%，而高表达EGFR的H1299细胞系的生长抑制率为0；在10 μΜ Erlotinib的浓度，H157细胞系的生长抑制率为24%，A549和Calu-1细胞系的生长抑制率分别约为10%和2%，H460和H1792的生长抑制率分别约为20%和21%，H1299的生长抑制率8%。上述结果表明，*EGFR*野生型NSCLC细胞系对Erlotinib耐药，且Erlotinib的细胞毒性与EGFR mRNA表达水平高低无关。

**2:**
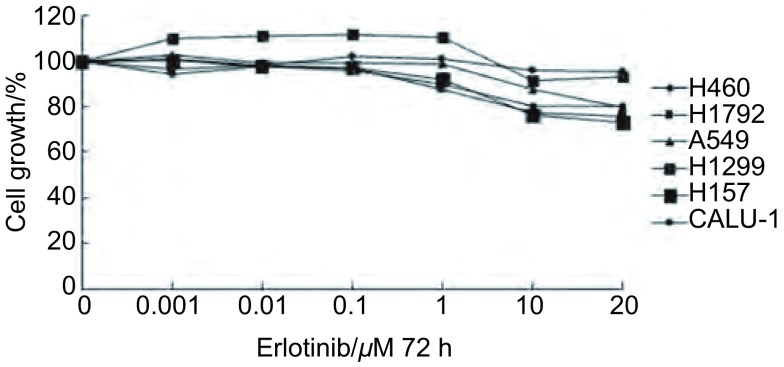
MTT方法检测*EGFR*野生型NSCLC细胞对Erlotinib药物敏感性 The measurement of cytotoxicity for *EGFR* wild-type NSCLC cells to Erlotinib by MTT assay

### 2.2 Erlotinib对*EGFR*突变型NSCLC细胞系毒性作用的研究

肺癌中*EGFR*突变多见于外显子18-21，即胞内酪氨酸激酶编码区，最常见突变形式包括外显子19的E746-A750del和外显子21的L858R点突变，这两种突变约占*EGFR*突变的85%-90%^[6]^，发生这两种突变的肿瘤细胞对EGFR-TKIs敏感，称为活化突变。部分肿瘤细胞可发生二次突变，最常见二次突变为外显子20的T790M突变，为耐药突变^[7]^。

H1650和HCC827细胞系均为EGFR外显子19的E746-A750del突变，H1975细胞系为外显子21 L858R点突变，但同时伴有外显子20的T790M二次突变。我们利用Western blot验证了上述3种NSCLC细胞系中*EGFR*的突变情况（图 3）。

**3:**
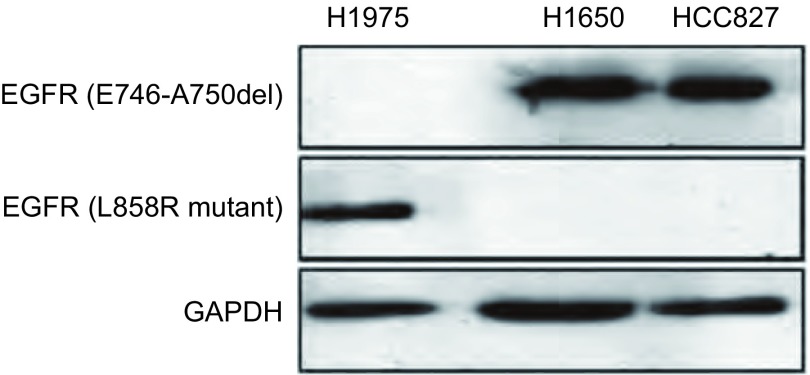
Western blot方法检测*EGFR*的突变情况 The *EGFR* mutations in H1975, H1650 and HCC827 cells

3株细胞系分别在7个浓度梯度Erlotinib的培养液中培养72 h，细胞毒性实验结果显示（图 4），Erlotinib能够明显抑制HCC827的生长，呈明显的浓度相关性，IC_50_为0.03 μΜ；H1975对Erlotinib高度耐药，这些结果均与预期的一致。但实验结果显示，H1650细胞系对Erlotinib相对耐药，约为HCC827的167倍，与预期的推测不一致。

**4:**
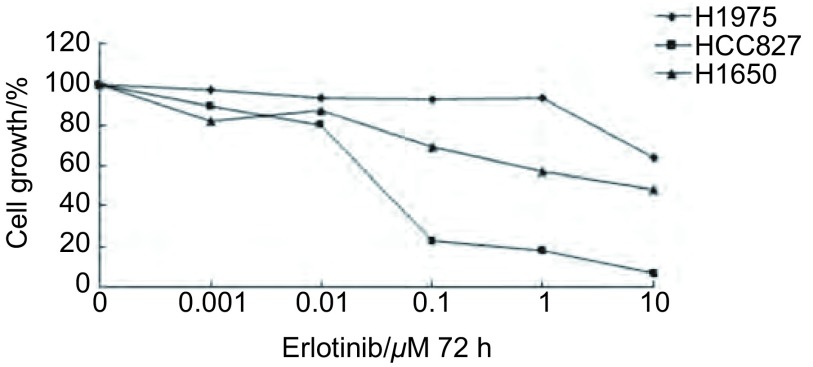
MTT检测*EGFR*突变细胞系对Erlotinib药物敏感性 The measurement of cytotoxicity for *EGFR*-mutant NSCLC cells to Erlotinib by MTT assay

### 2.3 Erlotinib对H1650细胞信号传导途径的影响

EGFR主要的信号传导通路包括：Ras/Raf/MEK/ERK通路和PI3K/AKT通路。EGFR与EGFR-TKI结合后，抑制EGFR本身磷酸化，从而抑制其下游相应的信号蛋白的磷酸化。

我们用10 μΜ Erlotinib处理HCC827和H1650细胞2 h，结果显示，在HCC827细胞中，AKT和ERK的磷酸化水平均明显下调（图 5），提示Erlotinib可以明显抑制其细胞内EGFR下游的信号通路；在H1650细胞中，p-ERK水平呈明显下降，但AKT的磷酸化水平无明显下降（图 5）。

**5:**
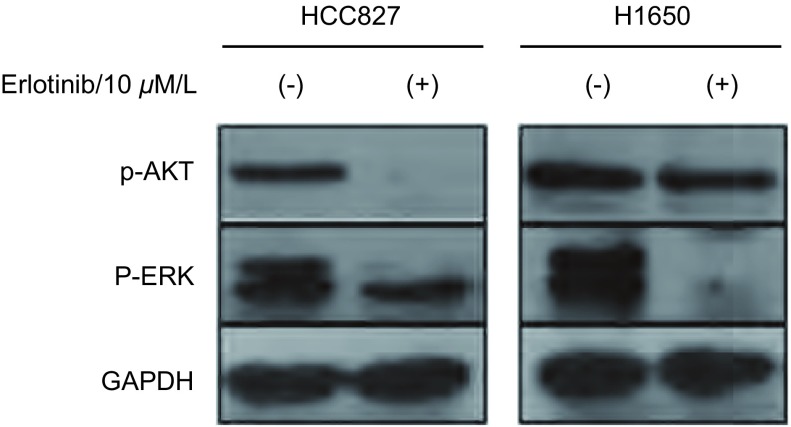
Western blot方法检测Erlotinib处理HCC827和H1650后p-ERK、p-AKT的表达水平 HCC827 and H1650 cells were treated with Erlotinib for 2 h, Anti-p-AKT and anti-p-ERK antibody was used to detect AKT and ERK phosphorylation, with GAPDH as the loading control.

Sos等^[8]^发现在H1650细胞中有PTEN的缺失。我们实验结果也证实了在H1650细胞中存在PTEN的缺失（图 6）。

**6:**
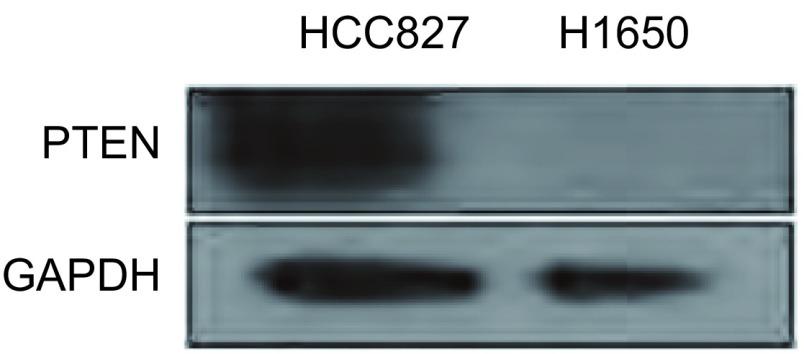
Western bot检测HCC827和H1650细胞中PTEN的表达水平 No PTEN protein detected in H1650 cells

蛋白酪氨酸磷酸酶（phosphatase and tensin homolog deleted on chromosometen, PTEN）是具有蛋白与脂质磷酸酯酶活性的双特异性磷酸酯酶，能特异地使磷脂酰肌醇-3, 4, 5-三磷酸3' 位脱磷酸，抑制Akt的磷酸化^[9]^。PTEN的缺失可以导致Akt的活化，从而可以解释Erlotinib为什么不能抑制H1650细胞AKT磷酸化水平，但可抑制ERK的磷酸化。为了进一步探讨H1650耐药与PTEN表达缺失的相关性，我们用10 μΜ LY294002（PI3K抑制剂）处理H1650及HCC827细胞2 h，进而检测p-AKT的水平，结果显示（图 7）HCC827细胞的p-AKT明显抑制，但对H1650细胞的p-AKT抑制作用不明显，进一步证实了因为PTEN的缺失，H1650的AKT磷酸化水平不受其上游调节；因为LY294002对Ras/Raf/MEK/ERK通路无作用，所以两种细胞系的p-ERK水平未被抑制（图 7）。

**7:**
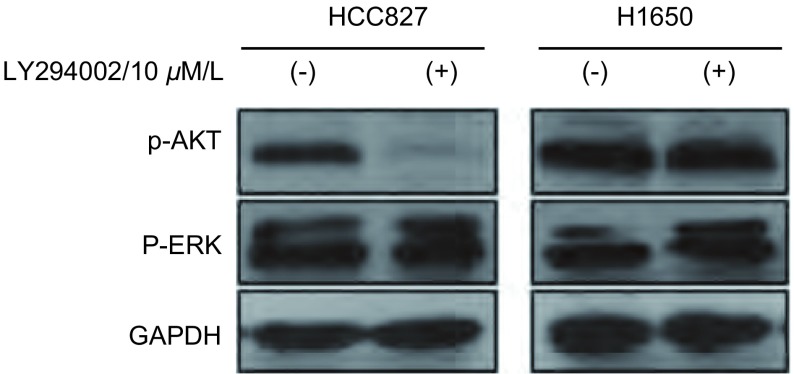
Western blot检测经LY294002处理后的HCC827和H1650细胞系的p-AKT、p-ERK表达水平 HCC827 and H1650 cells were treated with LY294002 (PI3K inhibitor) for 2 h, Anti-p-AKT and anti-p-ERK antibody was used to detect AKT and ERK phosphorylation, with GAPDH as the loading control.

综合上述结果，H1650细胞系对Erlotinib相对耐药可能与PTEN缺失导致AKT信号传导通路异常活化有关，而与Ras/Raf/MEK/ERK信号途径无关。

## 3 讨论

肺癌是死亡率最高的癌症之一，其中约80%-85%为NSCLC^[10]^，由于约70%的患者在就诊时已处于晚期，失去了手术的机会，且NSCLC对铂类等化疗药物的反应差，其5年生存率只有15%左右^[11]^。

EGFR是原癌基因*C-erbB1*的表达产物，属于酪氨酸激酶生长因子受体家族成员之一，EGFR主要的信号传导通路包括Ras/Raf/MEK/ERK通路和PI3K/AKT通路。在肿瘤细胞中，EGFR基因突变和扩增可使EGFR酪氨酸激酶不恰当激活，促进肿瘤的血管生成和肿瘤细胞的增殖、粘附、侵袭和转移。研究^[12]^显示，NSCLC伴转移的患者中，60%以上存在EGFR过度表达，且与这些患者的预后密切相关，因此以EGFR为靶点的抗癌治疗日益受到关注。*EGFR*突变主要发生在胞内酪氨酸激酶编码区，多发生于基因外显子18、19、20和21，与EGFR-TKIs敏感性相关的主要是位于外显子18、21的点突变和外显子19的缺失突变。其中，外显子21的点突变，使EGFR蛋白中该位点的氨基酸由亮氨酸转变为精氨酸（L858R）；外显子19第746-750位密码子的缺失（19 exon E746-A750del）导致EGFR蛋白中氨基酸序列丢失，改变了受体络氨酸激酶ATP结合槽的角度。

EGFR-TKIs通过与ATP竞争，结合于EGFR-TK胞内端的催化区域（EGFR结构域中高度保守的ATP结合位点），阻止EGFR的自磷酸化及下游的信号传导。EGFR-TKIs代表药物，Gefitinib或Erlotinib，作为二线药物已应用于标准化疗无效的NSCLC；对于存在*EGFR*突变的晚期NSCLC患者，EGFR-TKI一线化疗药物应用于临床已经得到了专家的共识^[13]^。但随着EGFR-TKI临床应用的日益增多，其耐药现象已成为临床工作中一大难题。引起耐药的原因多种多样，主要分为原发性和继发性耐药^[14]^，原发性耐药机制包括*KRAS*突变^[15]^；继发性耐药机制主要包括EGFR外显子20的T790M突变（苏氨酸转变为甲硫氨酸）和C-MET扩增两种，约占所有耐药机制的70%，尚有30%-40%患者耐药机制不清楚^[16]^。

本研究显示，*EGFR*野生型NSCLC细胞系对Erlotinib均耐药，且Erlotinib的药物敏感性与EGFR的mRNA表达水平无关，但与*EGFR*突变类型相关；具有EGFR外显子19缺失突变的H1650细胞对Erlotinib相对耐药。Guo等^[17]^发现在H1650细胞株中，PTEN蛋白表达缺失，因此对AKT的抑制作用消失。Yamamoto等^[18]^用Gefitinib处理PC-9细胞系（存在EGFR外显子19缺失突变）7个月后诱导出耐药细胞系，经验证发现，耐药细胞系是由于PTEN蛋白表达缺失导致对Gefitinib耐药的；他们用免疫组化的方法对4例Gefitinib获得性耐药的肺癌患者进行了治疗前后PTEN蛋白表达水平的比较，其中3例患者PTEN表达水平明显低于治疗前，考虑PTEN蛋白表达水平的缺失与Gefitinib获得性耐药有关。我们的实验结果也验证了在H1650细胞中存在PTEN蛋白表达的缺失，同时发现Erlotinib虽然可以抑制H1650细胞中的p-ERK水平，但p-AKT的水平无明显变化。因此推测，H1650对Erlotinib耐药可能与PTEN缺失有关。进一步应用PI3K抑制剂LY294002处理H1650细胞，发现其也不能抑制H1650细胞的p-AKT水平，进一步证实H1650细胞p-AKT水平不受其上游调节，其对Erlotinib相对耐药与PTEN表达缺失导致AKT信号传导通路异常活化有关，而与Ras/Raf/MEK/ERK信号途径无关。

此外，亦有文献报道H1650对Erlotinib的耐药可能与抑制BIM上调^[19]^和肿瘤细胞从上皮细胞向间充质细胞转化（epithelial mesenchymal transition, EMT）^[20]^有关，这些均有待于我们在未来的研究中进一步证实。
